# In Vitro Antimicrobial Activity of N-Acetylcysteine against Pathogens Most Commonly Associated with Infectious Keratitis in Dogs and Cats

**DOI:** 10.3390/antibiotics12030559

**Published:** 2023-03-11

**Authors:** Hanna Walter, Jutta Verspohl, Jessica Meißner, Hilke Oltmanns, Anna Karoline Geks, Claudia Busse

**Affiliations:** 1 Department of Ophthalmology, Small Animal Clinic, University of Veterinary Medicine Hannover, Foundation, 30559 Hannover, Germany; hanna.walter@tiho-hannover.de; 2 Institute for Microbiology, University of Veterinary Medicine Hannover, Foundation, 30173 Hannover, Germany; jutta.verspohl@tiho-hannover.de; 3 Department of Pharmacology, Toxicology and Pharmacy, University of Veterinary Medicine Hannover, Foundation, 30559 Hannover, Germany; jessica.meissner@tiho-hannover.de (J.M.); hilke.oltmanns@tiho-hannover.de (H.O.); 4 Department of Internal Medicine, Small Animal Clinic, University of Veterinary Medicine Hannover, Foundation, 30559 Hannover, Germany; anna.karoline.geks@tiho-hannover.de

**Keywords:** bacterial keratitis, N-acetylcysteine, antimicrobial, One Health, canine, feline

## Abstract

To determine the in vitro antimicrobial activity of N-acetylcysteine (NAC) against common pathogens associated with infectious keratitis in dogs and cats, clinical isolates of *Staphylococcus (S.) pseudintermedius* (n = 20), *Streptococcus (St.) canis* (n = 10) and *Pseudomonas (P.) aeruginosa* (n = 7) of canine and feline infectious ulcerative keratitis and a quality control strain (*P. aeruginosa* DSM 19880) were tested. The minimal inhibitory concentration (MIC) of NAC concentrations was determined using microdilution methodology. For *S. pseudintermedius* and *P. aeruginosa*, NAC concentrations in the range of 1.56 mg/mL (0.156%) to 100 mg/mL (10%), and for *St. canis*, concentrations ranging from 0.195 mg/mL (0.0195%) to 6.25 mg/mL (0.625%) were tested. For *S. pseudintermedius*, the MIC was 3.12 mg/mL (0.312%) for all tested isolates. For *P. aeruginosa* isolates and the quality control strain, the MIC ranged from 3.12 mg/mL (0.312%) to 6.25 mg/mL (0.625%). For *St. canis,* the MIC ranged from 1.56 mg/mL (0.156%) to 3.12 mg/mL (0.312%). NAC has an in vitro antimicrobial activity against three bacterial species commonly found in infectious keratitis in dogs and cats and therefore may be a promising alternative or adjuvant to topical antibiotics. The results warrant a clinical pilot study to assess the potential of NAC to reduce or replace the use of topical antibiotics in line with the One Health approach.

## 1. Introduction

Infectious ulcerative keratitis is a common and potentially vision- and globe-threatening disease in both animals and people [1,2,3]. Although interspecies and geographical differences exist, the most commonly found organisms in ulcerative keratitis in dogs, cats and horses are *Staphylococcus (S.)* species, *Pseudomonas (P.)* species and *Streptococcus (St.)* species [4,5,6,7,8,9,10,11,12,13,14,15]. In the study of Suter et al., *S.* species and *St.* species accounted for 66% of the isolates in dogs and 80% of the isolates in cats and horses [13]. Hewitt et al. found similar results in dogs from Midwestern America suffering from ulcerative keratitis, and the most common bacterial species in this study were *S. pseudintermedius* (26.7%), *St. canis* (12%) and *P. aeruginosa* (7.5%) [6]. In a feline study, *S.* species constituted 55% of total isolates [5]. Tsvetanova et al. showed that in canine melting corneal ulcerations, *P. aeruginosa* isolates were the most commonly found bacterial species (26/63) [16]. Melting corneal ulcers with pure *P. aeruginosa* infection were significantly more likely to have a poor outcome resulting in globe loss than with other cultures [16].

Antimicrobial treatment with topical medication is the therapy of choice in this infectious condition [17]. However, due to the different spectra of the activity of antibiotics, there is no antimicrobial agent available which is effective against all associated pathogens [4,5,6,7,9,12,13,14,18] and coinfections can occur [16]. Combination of different, mostly topically used antimicrobial agents is advised by some authors to treat the condition [19,20]. This can potentially lead to suboptimal efficacy of the agents since many antibiotics interfere with each other and even have an antagonistic effect [21]. In addition, topical antibiotic use bears a risk for the commensal flora of the ocular surface to shift towards pathogenic bacteria [22]. Furthermore, many topical antibiotics are epitheliotoxic and interfere significantly with corneal epithelial wound healing [23]. Even with intensive topical antimicrobial therapy, the prognosis for infectious ulcerative keratitis is guarded, with up to 57% of patients requiring surgical interventions [16]. This might be due to increasing resistances of the above-mentioned bacterial strains against antibiotics [4,5,6,7,9,12,13,14,24] but may, in the authors’ opinion, also reflect the late stage at which veterinary patients are presented at referral practices as well as certain predisposing anatomical features [25]. Therefore, many eyes have a poor visual outcome or even need to be enucleated, which makes the search for alternative or adjunctive treatment modalities necessary to improve the treatment success [26,27,28,29].

The increasing development of bacterial resistances further threatens the treatment success as well as dictating the cautious use of antimicrobials [12]. Various studies have shown that antimicrobial resistance in people, especially in pet owners and veterinarians, can be associated with the transmission of bacteria from canine origin [30,31,32,33]. Public health reasons dictate a reasonable and justified use of antimicrobials and preservation of reserve antibiotic agents for human medicine [30,31,32,33]. This drives the search for alternative substances with antimicrobial properties for the treatment of infections [34], which is especially important in veterinary medicine since the usage of antimicrobials in animals is becoming more and more regulated in the European Union (EU) [35].

N-acetylcysteine (NAC) is an acetylated form of the amino acid L-cysteine and an active precursor for the antioxidant glutathione [36]. For decades, NAC has been widely used in medicine for a variety of different indications. It is used as a potent mucolytic agent in chronic bronchitis, other pulmonary conditions [37] and keratoconjunctivitis sicca [38] as a potent chelating agent [39] protecting the liver and kidney from heavy metal poisoning; as an antioxidant, cytoprotectant and scavenger of reactive oxygen species in the context of clinical studies, animal experiments and cell culture experiments [40,41,42]; and it has anti-inflammatory properties by mediating cytokine release [43] and collagenase-inhibitor in corneal ulcerations [44,45].

NAC has been reported to be safe and well-tolerated at dosages up to 150 mg/kg in dogs and cats when given systemically [46,47]. Its bioavailability following oral or intravenous administration is very limited [48]; however, with topical application higher concentrations can be reached at the target tissue.

NAC has been used topically in human ophthalmology in the treatment of corneal wounds, chemical injuries, keratitis, dry eye disease and meibomian gland dysfunction [36]. The ophthalmic use of NAC in veterinary patients has also been investigated. Thermes et al. found that NAC can be safely used as an ocular treatment in the healthy eyes of rabbits in concentrations of 2.5% applied every five minutes for one hour [49]. A concentration of 20% applied three times within 15 min resulted in corneal and conjunctival irritations leading to a macroscopic redness of the conjunctiva [49]. In a masked randomized control trial in 20 New Zealand white rabbits, Fischak et al. showed a significantly faster corneal wound healing after epithelial debridement by manual scraping after the application of chitosan-N-acetylcysteine containing eyedrops twice daily [50].

In the study of Aldavood et al., 3% NAC resulted in significantly accelerated corneal wound healing in dogs [51]. Here, experimental corneal wounds (up to the depth of the anterior third of the stroma) were induced surgically in both eyes of 18 dogs. In six dogs, each one of the eyes was treated with 0.9% NaCl solution three times daily while the contralateral eye received 3, 10 or 20% NAC three times daily until healing. There was no drug toxicity seen in the investigated corneal samples when observed under a light microscopy [51].

NAC is commonly used as a collagenase inhibitor in melting corneal ulcers in veterinary ophthalmology [52]. It is a potent collagenase inhibitor and prevents or limits the destruction of corneal collagen by infectious organisms and inflammatory cells. Its inhibition of collagenases (e.g., matrix metalloproteinases 2 and 9) has been described at various concentrations (0.3–20%) in vitro and in vivo [52,53,54,55]. Kimmitt et al. showed that NAC at concentrations ≥0.5% is significantly more effective in inhibiting corneal degradation compared to the traditionally used allogenic or heterologous serum [52], which is time-consuming in its production, and its use is increasingly restricted by legal implications in the EU. Additionally, Ollivier et al. showed that 10% NAC resulted in an inhibition of the activity of matrix metalloproteinases in the tear film of horses with ulcerative keratitis by 98.8% [54]. NAC-containing eye drops at a concentration of 25 mg/mL are commercially available in the EU (N.A.C.^®^ collyre, TVM, Lempdes, France; Stromease TVM Tiergesundheit GmbH, Berlin, Germany).

In various studies, NAC has also been shown to have antimicrobial properties and to disrupt biofilm formation of bacterial origin in different bacterial species from various anatomical sites [56,57,58,59,60,61]. Bacteria of the ocular surface, e.g., *P.* species, are divided into nonpathogenic and pathogenic strains. Pathogenic pheno- and genotypes have been shown to lead to the invasion of corneal cells and concurrent corneal ulcerations [62]. Wang et al. also showed that the biofilm-formation ability, rates of virulence gene carriage and antibiotic resistance were higher in isolates of *S. pseudintermedius* from dogs with bacterial keratitis compared with those isolated from healthy dogs [63]. Studies investigating the effect of NAC on these pathogenic bacterial strains causing corneal ulcerations are lacking.

Therefore, the objective of this study was to investigate the in vitro antimicrobial activity of NAC on pathogens most commonly associated with infectious ulcerative keratitis in dogs and cats. We hypothesized that in addition to the collagenase inhibition [52,53,54,55] and disruption of the biofilms [57,58,60,61], NAC has antimicrobial properties. This may be valuable in the treatment of infectious ulcerative keratitis or as a prophylactic agent to prevent corneal infection in noninfected corneal ulcerations.

## 2. Results

NAC had an in vitro antimicrobial effect against all 38 bacterial isolates tested (37 clinical isolates and 1 quality control strain).

For *S. pseudintermedius*, the MIC was 0.312% for all tested isolates. In seven *S. pseudintermedius* isolates, visual inspection showed no button formation but turbidity compared to the negative growth controls. In one case, visual turbidity was inconclusive and, therefore, 100 µL of the solution was applied on a Sheep blood agar overnight, which showed bacterial growth so that the next higher concentration that showed a clear fluid was documented as MIC.

For *St. canis*, the MIC ranged between 0.156% (1.56 mg/mL) and 0.312% (3.12 mg/mL). In one testing cycle, two *St. canis* isolates had a MIC of 0.312%; in all other cycles of *St. canis* isolates, the MIC was 0.156%.

The MIC of *P. aeruginosa* isolates ranged from 0.312% (3.12 mg/mL) to 0.625% (6.25 mg/mL), respectively. In one testing cycle, one *P. aeruginosa* isolate had a MIC of 0.625%; in all other cycles of *P. aeruginosa* isolates, the MIC was 0.312%.

One *P. aeruginosa* isolate was of feline origin, the quality control strain was of human origin and all other tested isolates were of canine origin.

Consistent bacterial growth was seen in all of the positive growth control wells, and there was no growth in the negative growth control wells.

## 3. Discussion

In recent years, researchers from different medical fields (dermatology, orthopedics, dentology) have shown that NAC has a potent antimicrobial effect and can disrupt biofilm formation [56,57,58,59]. The proposed modes of antimicrobial actions of NAC are a competitive inhibition of the uptake of amino acids, such as cysteine, by bacterial cells or the reaction of its own sulfhydryl group with the bacterial cell proteins [64,65]. Human, canine and feline infectious ulcerative keratitis have also been associated with the biofilm formation of different microorganisms [63,66,67,68]. The complex bacterial communities are encapsulated in an extracellular polymeric matrix, and hereby, they have an increased resistance to antibiotics and the host immune response, increasing bacterial survival, which further complicates the treatment of bacterial keratitis [69]. The results of our in vitro study show an antimicrobial property of NAC against pathogens associated with bacterial keratitis isolates from dogs and cats at a relatively low concentration (0.156–0.625%, 1.56–6.25 mg/mL) and well below the concentrations used for its anti-collagenase properties. It is noteworthy that all tested methicillin-resistant *S. pseudintermedius* isolates were susceptible to 0.312% NAC. Chan et al. investigated NAC in ranging concentrations on pathogens commonly associated with canine otitis externa [56]. For *S. pseudintermedius* and *P. aeruginosa* (including the quality control strain), they found a MIC range of 0.25 to 0.5% (2.5–5 mg/mL). For β-hemolytic *Streptococci*, they detected a MIC of 1.0% (10 mg/mL) [56]. The results of our study are in part comparable to the results of Chan et al. when looking at the quality control strain as well as the *Staphylococcus* and *Pseudomonas* isolates [56]. However, the tested *St. canis* isolates in our study were more susceptible to NAC, resulting in a MIC range of 0.156 to 0.312% (1.56–3.12 mg/mL). This may be explained by the different anatomical locations the bacteria originated from (canine otitis externa vs. canine bacterial keratitis).

Moon et al. investigated the antibacterial and biofilm-disrupting properties of ranging NAC concentrations on endodontic pathogens. The authors found an antibacterial activity with a MIC range of 0.78–1.56 mg/mL as well as an inhibition of biofilm formation by monospecies and multispecies bacteria (*Actinomyces naeslundii, Lactobacillus salivarius, Enterococcus faecalis, Streptococcus mutans*) at a MIC range of 0.78–3.13 mg/mL [59]. Preformed mature multispecies biofilms were disrupted within 10 min using treatment with NAC at concentrations of 25 mg/mL or higher [59]. These findings are particularly important in bacterial keratitis patients of varying species since the causative bacterial isolates are known to colonize the corneal surface as biofilm populations [69]. Since NAC has biofilm-disruptive properties, it could possibly improve the efficacy of antibiotics used in this disease. Olofsson et al. showed that NAC inhibited bacterial growth in various concentrations as well as decreased bacterial adhesion and even detached bacteria that were adhering to stainless steel surfaces [70]. The results further indicated that NAC reduces the production of extracellular polysaccharides in most tested bacteria, even at concentrations at which bacterial growth was unaffected [70]. NAC had a potent in vitro antimicrobial effect on all investigated bacterial species in the presented study, and since this is in line with the results of other specialties, NAC seems to be a promising antimicrobial agent.

Many studies have investigated the synergistic or antagonistic effect of NAC when combined with an antibiotic agent and have found different results. Onger et al. investigated the effect of NAC and ciprofloxacin alone or combined on *P. aeruginosa* biofilm in infected bone cement [60]. Their results showed that the combination of NAC and ciprofloxacin enhanced the beneficial effect of ciprofloxacin in bone cement. In contrast, Goswami and Jawali showed that the MICs of ciprofloxacin and ofloxacin increased substantially for *Escherichia coli* and *Klebsiella aerogenes* strains and moderately for *P. aeruginosa* in the presence of 1.632 mg/mL NAC [71]. In contrast, in the same study, NAC did not alter the MICs of chloramphenicol and tetracycline for any bacterial strain, and the authors suggested that the action of these antibiotics is not affected by the presence of NAC [71]. Parry and Neu also showed an inhibition in the growth of both Gram-negative and Gram-positive bacteria when exposed to 2–20 µg/mL NAC [72]. They further detected a synergistic effect from NAC and carbenicillin or ticarcillin in the inhibition of *P. aeruginosa*. However, they also detected an antagonistic effect when NAC was combined with gentamicin or tobramycin [72]. Since NAC is often combined with an antibiotic agent in patients suffering from bacterial keratitis, future studies should address the potential synergistic or antagonist effect of NAC when combined with commonly used topical antibiotics on the bacterial growth of pathogenic bacteria of the ocular surface.

The limitations of this study include its in vitro nature as results may differ from the in vivo situation due to factors such as the contact time of the isolates with NAC in the 96-well-plates compared to the expected contact time when NAC is applied to the ocular surface, dilution of NAC due to tear production or ocular clearance [73]. A further limitation is the relatively small sample size and that the majority of isolates were of canine origin.

Future research should address the interference of antibiotic agents and NAC, the extent and time-dependent tissue concentration of NAC in the cornea and if a time lag between applications to the ocular surface may allow overcoming this potential disadvantage or whether a combination with antibiotics needs to be carefully chosen. In dogs and cats, topical ophthalmic therapeutics usually remain on the ocular surface for 5–10 min before they are cleared through the nasolacrimal duct and spillage over the lower eyelid [74]. Therefore, the relevance of contact time on the antimicrobial effect of NAC should be evaluated before in vivo studies in dogs and cats are conducted. Time-to-killing assays are planned to investigate the time dependence of this process. It could be advantageous to increase the contact time of NAC with the ocular surface to improve its effect and potentially reduce the application frequency to the eye. Recently, NAC has been combined with chitosan, which is a biopolymer that forms a protective glycocalyx layer on the ocular surface [50] and can have up to 24 h ocular surface retention time. The mucoadhesive properties of chitosan are enhanced with the addition of thiol groups from NAC, due to the formation of disulfide bonds between the thiol groups and cysteine-rich domains of corneal mucus glycoproteins, leading to the stabilization of chitosan–NAC on the ocular surface [75]. Results of clinical studies have shown that topical chitosan–NAC used once to twice daily significantly increases the mean tear film thickness and improves corneal integrity in human patients with dry eye syndrome [76,77,78]. A combination of NAC with chitosan may therefore be a potent possibility. Further, this combination has also been shown to function as a nanocarrier system resulting in increased trans-corneal penetration, prolonged precorneal retention and enhanced ocular bioavailability [79,80]. The potential development of bacterial resistances against NAC, the effect on commensal ocular surface flora and the effect of 0.625 to 3% NAC solutions on corneal cells and wound healing necessitate further investigations.

## 4. Materials and Methods

### 4.1. Bacterial Isolates

Clinical isolates of canine and feline infectious keratitis were collected by a diagnostic laboratory (LABOKLIN, Bad Kissingen, Germany). Submitted samples comprised corneal swaps of infected corneal ulcerations, as documented by the submitting veterinary surgeon. Isolates were specified with matrix assisted laser desorption ionization-time of flight mass spectrometry (MALDI-TOF) mass spectrometry. A resistance testing against commonly used antibiotics was performed. Investigated bacteria included *S. pseudintermedius* (n = 20), *St. canis* (n = 10) and *P. aeruginosa* (n = 7), and a control strain (*P. aeruginosa* DSM 19880) was tested. Three isolates of *S. pseudintermedius* were methicillin and multidrug-resistant strains.

### 4.2. Minimal Inhibitory Concentration (MIC) Testing

Sterile cation-adjusted Mueller Hinton Broth (CAMHB; Mueller-Hinton-Bouilllon, Carl Roth GmbH + Co. KG, Karlsruhe, Germany) and the pure substance of NAC (N-Acetyl-L- cysteine, SIGMA-ALDRICH CHEMIE GmbH, Steinheim, Germany) were used to create stock solutions for *S. pseudintermedius* and *P. aeruginosa* testing as follows: 1.56 mg/mL (0.156%), 3.12 mg/mL (0.312%), 6.25 mg/mL (0.625%), 12.5 mg/mL (1.25%), 25 mg/mL (2.5%), 50 mg/mL (5%) and 100 mg/mL (10%).

In a separate experiment, *St. canis* isolates were tested. Since these are fastidious bacterial species, equine lysed blood was initially added to the NAC solutions. However, with this approach MICs could not be evaluated due to the brown staining of the solution after incubation which prohibited the evaluation. To overcome this problem, NAC solutions for *St. canis* testing contained a small amount of inactivated chicken serum (115 µL serum in 11.5 mL CAMHB). In a preliminary experiment, *St.* isolates were tested with the above-mentioned stock solutions. NAC concentrations above 0.625% showed no bacterial growth. Therefore, created stock solutions for *St. canis* testing were chosen as follows: 0.195 mg/mL (0.0195%), 0.39 mg/mL (0.039%), 0.78 mg/mL (0.078%), 1.56 mg/mL (0.156%), 3.12 mg/mL (0.312%) and 6.25 mg/mL (0.625%).

All stock solutions were prepared freshly on each day of MIC testing. The antimicrobial susceptibility of each isolate was determined using MIC microdilution methodology as recommended by the Clinical and Laboratory Standards Institute.

**Figure 1:**
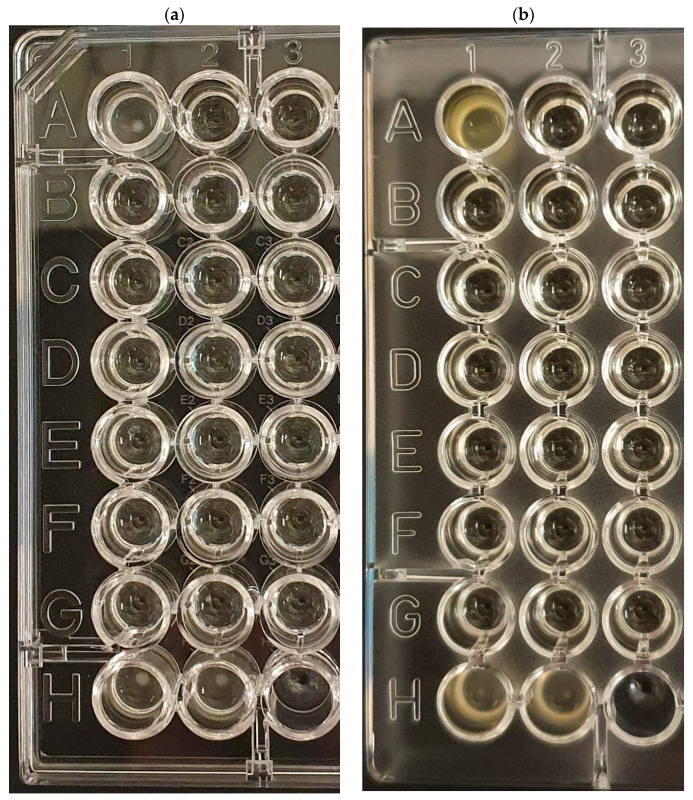
This figure shows part of a round-bottom 96-well plate of the NAC concentrations in varying concentrations, positive and negative growth controls. Columns 1 A to G are the NAC concentrations with bacterial suspension added with increasing concentrations beginning from A1 comprising 0.156% to G1 comprising 10% NAC. H1 and H2 show the two positive growth controls and columns 2 and 3 A to G are two negative growth controls per NAC concentration. H3 is empty. (**a**) Shows a *Staphylococcus pseudintermedius* isolate, showing button formation indicating bacterial growth at A1, H1 and H2 (positive growth controls). The next higher concentrations of NAC (B1–G1) and the negative growth controls (A2 and 3-G2 and 3) show a clear fluid, and hence, no bacterial growth. (**b**) Shows a *Pseudomonas aeruginosa* isolate where bacterial growth can be detected as turbidity formation, shown in A1, H1 and H2 (positive growth controls). The next higher concentrations of NAC and the negative growth controls (A2 and 3-G2 and 3) show a clear fluid, and therefore, no bacterial growth.

Bacterial isolates were cultured on 5% Sheep Blood Columbia Agar (COLUMBIA AGAR WITH SHEEP BLOOD, Oxoid Deutschland GmbH, Wesel, Germany) and incubated overnight at 37 °C. A bacterial suspension for each isolate was prepared in sterile saline (NaCl 0.9%, B. Braun Medical AG, Sempach, Switzerland) and adjusted to 0.5 McFarland standard. Then, 20 µL of the bacterial inoculum was added to the 180 µL NAC solution to achieve the same amount of colony-forming units (cfus)/mL per isolate. All clinical isolates were tested in triplicate, the quality control strain was tested in duplicate. Two negative growth controls per NAC concentration containing only CAMHB (20 µL) and NAC (180 µL) and two positive growth controls per isolate containing CAMHB (180 µL) and bacterial suspension (20 µL) were tested. The round-bottom 96-well plates (CELLSTAR®, 96 Well Cell Culture Plate, sterile, U-bottom, with lid, Greiner Bio-One GmbH, Frickenhausen, Germany) were evaluated with a visual assessment for whether there was button or turbidity formation after overnight (16–20 h) incubation at 37 °C (see Figure 1).

The Minimal Inhibitory Concentration was defined as the NAC concentration that completely inhibited growth, which was seen as no turbidity or button formation, resulting in a clear fluid. Results of each 96-well plate were photographically documented. MIC ranges (minimum and maximum) were recorded for each isolate. As previously recommended [81], MIC50 and MIC90 were not calculated due to the low number of tested isolates.

### 4.3. Data Analysis

The statistical analysis comprised a descriptive analysis (Excel 2019, Version 1808, Microsoft Office Professional Plus 2019) of the collected data.

## 5. Conclusions

We conclude that N-acetylcysteine has a potent in vitro antimicrobial effect against *Staphylococcus pseudintermedius*, *Streptococcus canis* and *Pseudomonas aeruginosa* isolates which are the most commonly found bacteria in infectious ulcerative keratitis in dogs and cats. If in vivo studies further confirm our results, then NAC may have a role as a topical ocular antimicrobial or adjuvant to other antimicrobial agents. It could aid in the reduction of antibiotic consumption in line with the One Health approach, as well as improve the treatment efficacy in patients with infectious keratitis.

## Data Availability

All necessary data are found within the text of this manuscript.
